# Global Distribution and Dispersal Pathways of Riparian Invasives: Perspectives Using Alligator Weed (*Alternanthera philoxeroides* (Mart.) Griseb.) as a Model

**DOI:** 10.3390/plants15020251

**Published:** 2026-01-13

**Authors:** Jia Tian, Jinxia Huang, Yifei Luo, Maohua Ma, Wanyu Wang

**Affiliations:** 1 Chongqing Institute of Green and Intelligent Technology, Chinese Academy of Sciences, No. 266 Fangzheng Ave., Shuitu Town, Beibei District, Chongqing 400714, China; tianjia@cigit.ac.cn (J.T.); luoyifei25@mails.ucas.ac.cn (Y.L.); mamaohua@cigit.ac.cn (M.M.); wangwanyu21@mails.ucas.ac.cn (W.W.); 2 University of Chinese Academy of Sciences, No. 1 Yanqihu East Rd., Huairou District, Beijing 101408, China

**Keywords:** *Alternanthera philoxeroides* (Mart.) Griseb., riparian habitats, biological invasion, dispersal pathways, multiple scales, riverscape management

## Abstract

In struggling against invasive species ravaging riverscape ecosystems, gaps in dispersal pathway knowledge and fragmented approaches across scales have long stalled effective riparian management worldwide. To reduce these limitations and enhance invasion management strategies, selecting appropriate alien species as models for in-depth pathway analysis is essential. *Alternanthera philoxeroides* (Mart.) Griseb. (alligator weed) emerges as an exemplary model species, boasting an invasion record of around 120 years spanning five continents worldwide, supported by genetic evidence of repeated introductions. In addition, the clonal reproduction of *A. philoxeroides* supports swift establishment, while its amphibious versatility allows occupation of varied riparian environments, with spread driven by natural water-mediated dispersal (hydrochory) and human-related vectors at multiple scales. Thus, leveraging *A. philoxeroides*, this review proposes a comprehensive multi-scale framework, which integrates monitoring with remote sensing, environmental DNA, Internet of Things, and crowdsourcing for real-time detection. Also, the framework can further integrate, e.g., MaxEnt (Maximum Entropy Model) for climatic suitability and mechanistic simulations of hydrodynamics and human-mediated dispersal to forecast invasion risks. Furthermore, decision-support systems developed from the framework can optimize controls like herbicides and biocontrol, managing uncertainties adaptively. At the global scale, the dispersal paradigm can employ AI-driven knowledge graphs for genetic attribution, multilayer networks, and causal inference to trace pathways and identify disruptions. Based on the premise that our multi-scale framework can bridge invasion ecology with riverscape management using *A. philoxeroides* as a model, we contend that the implementation of the proposed framework tackles core challenges, such as sampling biases, shifting environmental dynamics, eco–evolutionary interactions using stratified sampling, and adaptive online algorithms. This methodology is purposed to offer scalable tools for other aquatic invasives, evolving management from reactive measures to proactive, network-based approaches that effectively interrupt dispersal routes.

## 1. Introduction

Biological invasions are profoundly reshaping ecosystems worldwide [1,2]. Although risk assessments and management strategies increasingly emphasize prevention and rapid response, their effectiveness depends on a detailed comprehension of how alien species spread across time and space [3,4]. Identifying the primary dispersal pathways (including natural and human-mediated pathways) remains pivotal for targeting surveillance, closing introduction pathways, prioritizing biosecurity measures at high-risk nodes (e.g., ports, inter-basin transfer points), and designing interventions with maximum leverage [5]. Yet, despite decades of research, our grasp of global dispersal pathways remains incomplete, with evidence scattered across disparate data sources that vary in standards, spatial scope, and observational biases [6,7]. Furthermore, invasion biology, unlike classical model-organism research, has often been studied case-by-case, limiting cumulative methodological progress [8].

Such research fragmentation is especially evident in the studies of riparian invasives, where analytical approaches are often isolated. Often, the common correlative niche models rarely account for river network dynamics, and the network-based diffusion models ignore hydrodynamic constraints and propagule biology. Genomic source-attribution studies can resolve introduction histories yet struggle to connect historical records to real-world transport routes along river systems [9]. Consequently, this yields an incomplete view that hampers precise policy and management in riverine landscapes. A promising solution involves developing a model system that is both ecologically representative and methodologically tractable, by which a riparian alien species whose invasion history, life history, and data availability allow rigorous, multi-source integration and hypothesis testing.

Therefore, an effective invasive model shall catalyze standardization in data architectures, enable benchmarking of diverse analytical methods, and evaluate generalizability across regions and management contexts [10], particularly in the river ecosystems. To achieve this, such a model should meet these key criteria: (1) broad and well-documented global distribution with multiple independent introductions; (2) clear human-mediated and natural dispersal components enabling pathway disaggregation; (3) strong ecological impacts that motivate translational management research; (4) rich, multi-scale datasets (genomic, ecological, remote sensing, trade/traffic); and (5) biological traits that link mechanism to spread (e.g., propagule type, survival thresholds).

Among the riparian alien species, *Alternanthera philoxeroides* (Mart.) Griseb. (Alligator Weed) appears to fulfill the criteria and provides unique advantages as an ideal model for global invasion pathway studies. It has a widespread distribution across subtropical–temperate zones, with repeated introductions from its South American origins to North America, Australia, Asia, Europe, and Africa [11,12]. Its multi-introduction history and clonal propagation [13,14,15] create a natural lab for analyzing source–sink dynamics, founder effects, and the balance between human-mediated and hydrological dispersal. The species relies on vegetative fragments that endure transport and readily re-root [16,17,18], linking spread to hydrodynamics, floods, and human vectors [19]. In addition, its phenotypic plasticity and amphibious adaptability [20,21] enable testing for global model species across aquatic–terrestrial gradients and disturbances. The species’ properties also drive significant ecological and economic impacts, bridging mechanisms to management evaluations at both local and global scale [22,23,24]. Moreover, it can benefit from diverse datasets, including population genetics/genomics [15], remote-sensing dynamics [25], eDNA (environmental DNA) detection [26], hydrological transport models [27], and trade corridor mappings [28]. However, while much research has covered the traits of *A. philoxeroides* and management well, it still lacks systematic analysis of its role in multi-scale invasion networks and the coupling of evolutionary processes with invasion pathways. Thus, combining phylogenetics, population genetics, and pathway data is essential to reveal how evolutionary divergence affects pathway selection and spread, enhancing risk assessment and interventions in riverscape management.

Hence, the key motivation of this review is to synthesize *A. philoxeroides*’ global distribution and pathways, assess methodological advances, and identify priorities to address gaps in riparian alien species studies. The research questions are as follows: (1) What is *A. philoxeroides*’ potential as a global model for predicting riparian invasion patterns, using its distribution, genetics, and mechanistic data? (2) How can an integrated system, from biological mechanisms to real-time monitoring, forecast and manage risks under environmental changes? In this review, we propose an integrative framework using *A. philoxeroides* as a model riparian alien species in river ecosystems. This framework enables a multilayer pathway inference approach, integrating population genomics for source tracking, hydrodynamic and physiological factors for propagule viability, network analytics for shipping, road, and inter-basin systems, and causal inference for policy/infrastructure impacts. Beyond species-specific findings, this framework can also test transferable workflows, including data standards, analytical pipelines, and validation protocols, for other plant invaders and aquatic/semi-aquatic taxa with mixed dispersal vectors.

## 2. The Potential of *A. philoxeroides* as a Model

### 2.1. Long History of Global Invasion Patterns

**Figure 1:**
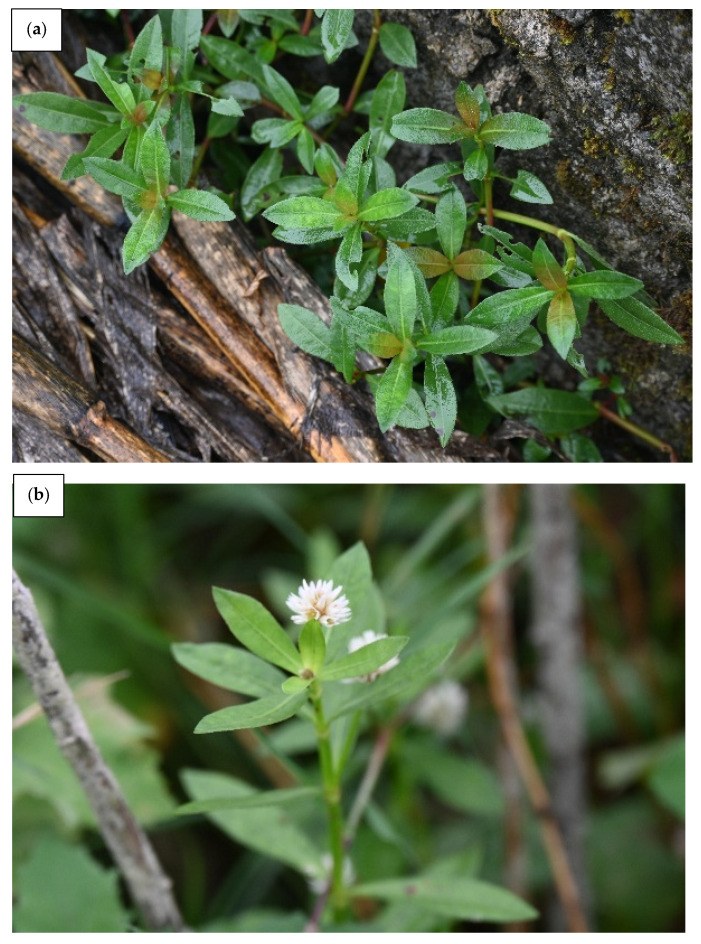
Photos of *A. philoxeroides* in flowering (**a**) and vegetative stage (**b**), which were shot by the authors along the riparian zone of China’s Yangtze River.

**Figure 2:**
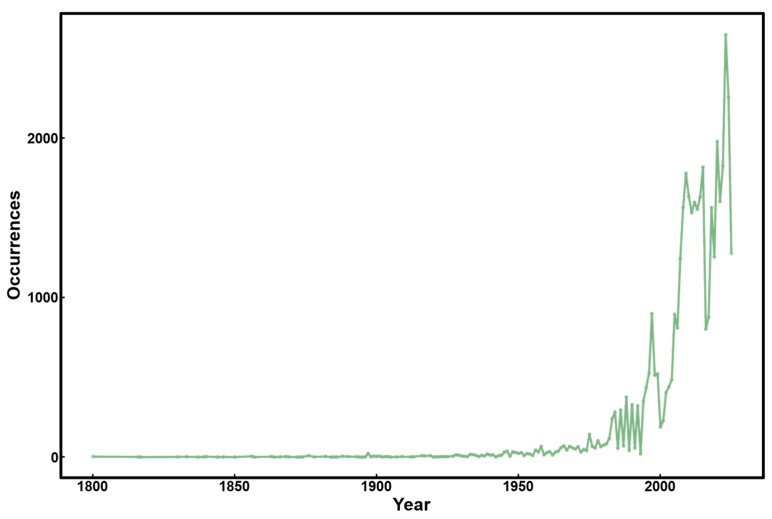
Occurrence records of *A. philoxeroides* per year from 1800 to 2025 (data is derived from Global Biodiversity Information Facility, GBIF [48]).

**Figure 3:**
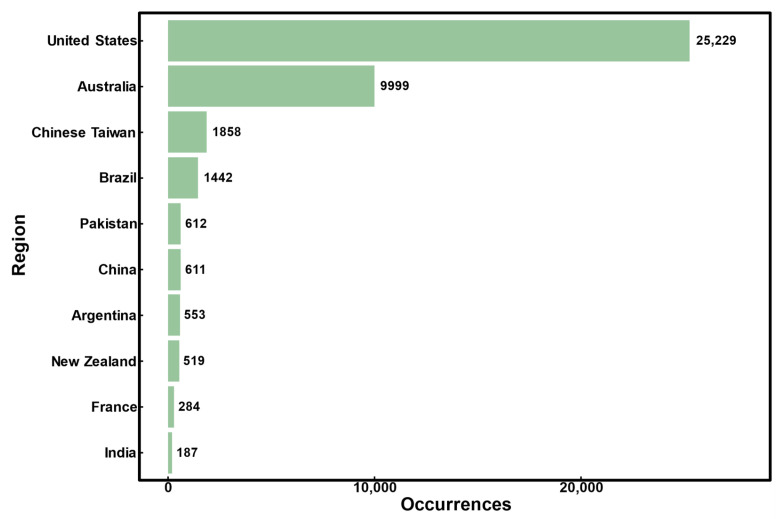
Occurrence records of *A. philoxeroides* observed in the top 10 countries (data is derived from GBIF [48]).

*A. philoxeroides*, an amphibious herbaceous plant native to South America, has been documented from southern Brazil southward to approximately 39° S (Figure 1). The earliest confirmed introduction outside its native continent occurred in 1897 in Mobile, Alabama, USA [29,30]. Through the twentieth century, multiple introductions, primarily via ornamental and aquascaping trade, shipping, and inter-basin water transfers, facilitated rapid naturalization across various continents during invasion history (Figure 2 and Figure 3). It is commonly known as alligator weed, gator grass, pigweed, or red legs, underscoring its widespread recognition in the invaded areas.

Up to now, *A. philoxeroides* has established in subtropical to warm-temperate zones across Asia, Europe, North America, South America, and Oceania, causing negative effects in over 30 countries [12,31] (Table 1). For instance, in China, the species has expanded significantly in the Yangtze River Basin, shifting northward from about 21.5° N to 36.8° N in recent decades and harming biodiversity, water resources, and agriculture [32,33]. Australia had designated the weed of National Significance, with severe impacts in New South Wales and Queensland, including wetland degradation and disrupted water flow [34,35]. In the United States, *A. philoxeroides* is prevalent in southern states like Florida, Louisiana, and Texas, as well as California, where it diminishes native plant diversity, modifies aquatic habitats, and hinders water management [15]. In Europe, populations of *A. philoxeroides* are widely established in France (particularly the Mediterranean) and Italy, with potential for further spread to adjacent countries, highlighting the need for vigilant monitoring and control [36,37].

Invasion success of *A. philoxeroides* arises from the interplay of propagule traits, human-assisted dispersal, and ecological prowess, fueling its global expansion for over 125 years and sustaining persistent infestations across decades. The species reproduces mainly through clonal fragments that re-root easily from tiny pieces, enabling rapid spread in waterbodies and riparian zones with minimal sexual seed production [17,20,38,39], amplified by human vectors like hull/gear contamination, canals, dredge spoil, inter-basin transfers, farm equipment, and flood debris for long-distance dispersal [38]. Once established, dense mats from rapid growth outcompete natives, simplify habitats, and disrupt nutrients and oxygen via shading and organic buildup, especially in eutrophic waters [38,40], further boosted by enemy-release from co-evolved predators [41]. This long-term dominance of the propagule trait is also supported by broad environmental tolerance, with optimal growth at 30–37 °C (peak photosynthesis) and 30 °C (shoot emergence), persistence down to 10–20 °C annual means, thriving in pH 4.8–7.7, moderate-salinity (10–30% seawater), and nutrient-rich conditions [42,43,44,45,46,47] (Table 2).

**Table 1:** Distribution and reported records of *A. philoxeroides* in representative countries.

Country/Territory	Origin	Distribution	Reported Records	Reference
Argentina	Native	Present, widespread	original	[46]
Brazil	Native	Present, widespread	original	[46]
Mexico	Introduced	Present	2012	[49]
United States	Introduced	Present, restricted	1897	[29,30]
France	Introduced	Present	1971	[50]
Spain	Introduced	Present, restricted	2015	[51]
Italy	Introduced	Present	2001	[36]
China	Introduced	Present	1892	[52]
Chinese Taiwan	Introduced	Present, widespread	1934	[53]
India	Introduced	Present, widespread	1965	[54]
Japan	Introduced	Present	1989	[55]
Pakistan	Introduced	Present	2014	[56]
Singapore	Introduced	Present	1953	[57]
Australia	Introduced	Present, widespread	1946	[58]
New Zealand	Introduced	Present, widespread	1906	[59]

**Table 2:** Suitability of environmental conditions for *A. philoxeroides*.

Environment Type	Suitability	Example Regions	Reference
Temperate regions (optimal 15–30 °C)	More strongly invasive	Southeastern United States, Riverina (Australia)	[42,60]
Tropical and subtropical regions	Suitable	Northern Argentina, Southern Brazil	[61]
Aquatic systems (rivers, lakes)	Suitable	Mississippi River (USA), Lake Ohakuri (New Zealand)	[61]
Terrestrial systems (croplands, riparian zones)	Suitable	Agricultural lands in Eastern China, riparian zones in New South Wales (Australia), Sacramento–San Joaquin Delta (California, USA)	[62,63]
Regions with occasional frost	Tolerated/Can survive	Northern China plain, highlands of South Africa	[64,65,66]

### 2.2. Broad-Scale Dispersal and Establishment from Propagules to Networks

In particular, *A. philoxeroides* distinguishes itself as a global model species by dispersing and establishing populations via a multi-scale array of mechanisms, starting from individual propagules and scaling up to self-sustaining riverscape networks. As vegetative propagules are the central to its invasion success [17,60,67,68,69], hydrochory plays a key role in this dispersal process, with fragments often dislodged by flow surges, tidal currents, boat wakes, mowing, or herbivory [38,70,71], then carried downstream to lodge along riverbanks, floodplains, and backwaters. During high-flow events, flood rafting further extends dispersal by forming floating mats of *A. philoxeroides* population that can drift tens to hundreds of kilometers before settling, especially in expansive floodplains with prolonged hydraulic retention [72,73].

In addition to unintentional drift, anthropogenic vectors are the key drivers, as fragments adhere to boat hulls, trailer bunks, propellers, fishing nets, and personal watercraft [74]. They also tangle in intake grates, cling to waders, and accumulate on dredging equipment, enabling cross-basin dispersal that natural connectivity would otherwise preclude [75]. Although *A. philoxeroides* is largely clonal in many invaded regions with low seed set under typical conditions [17,39], seed production can occur in certain climatic envelopes, cytotypes, or hybrid backgrounds, contributing a cryptic seed rain that augments propagule pressure and enhances the probability of long-distance establishment via sediment-borne diaspores [76]. In the regions where sexual reproduction is functional, seed banks of *A. philoxeroides* may form shallow, transient reservoirs that complement vegetative spread, especially after disturbances that expose bare substrate [77].

**Figure 4:**
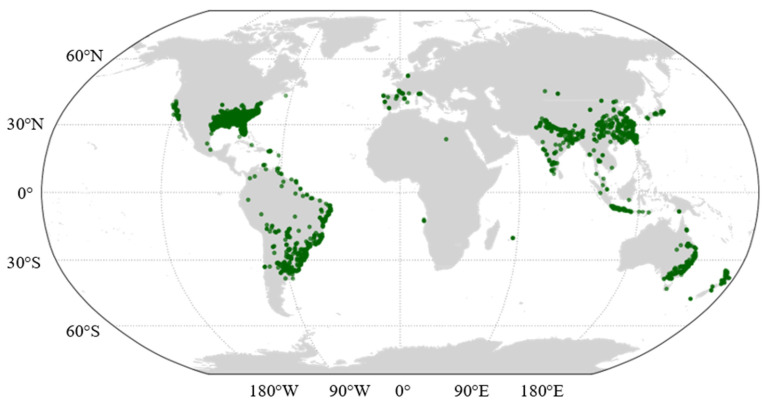
Global distribution of *A. philoxeroides* (data is derived from GBIF [48]).

Furthermore, human-mediated dispersal pathways form an extensive lattice that propels *A. philoxeroides* from isolated propagules to expansive riverine networks [78,79], leading to the global distribution of the species (Figure 4). In particular, the ornamental trade and aquascaping inadvertently facilitate spread through mislabeling, shipment contamination, or similar taxa in mixed consignments [80], while inter-basin water transfers and canal systems create persistent corridors across watersheds via flow reversals, locks, and drawdowns [20]. In addition, agricultural landscapes further enable redistribution along ditches, canals, and field edges [81], augmented by road, rail, shipping networks, and post-disaster activities like dredging and spoil placement. Though direct evidence is sparse, these mechanisms likely drive secondary spread and merit deeper study.

Upon arrival, establishment of *A. philoxeroides* is shaped by broad-scale habitat filtering and priority effects [33], with hydrodynamics favoring rooting in low-energy zones (e.g., eddies, oxbows) where reduced shear and extended residence times prevail. Fine sediments, nutrient enrichment from runoff [82], and stable temperature/photoperiod conditions enhance its colonization, though fluctuations can aid or hinder it [83]. Dense mats of *A. philoxeroides* then suppress natives, reduce diversity, and induce hypoxia, altering ecosystem structures at riverscape scales [33].

Once established, biotic interactions further amplify broad-scale persistence and expansion of *A. philoxeroides*. Competition with macrophytes and algae reshapes communities and nutrient cycles, while herbivores yield variable effects modulated by aquatic environments [84,85]. Pathogens induce dieback mitigated by clonality, pollinators enable rare sexual reproduction and potential hybridization, and rhizosphere microbiomes boost tolerance, nutrient uptake, and allelopathy [86,87]. Together, these processes foster networked invasion dynamics, including hydrological/human corridors disperse propagules, and the established patches along riparian habitats reinforce metapopulation of the species across riverscapes.

### 2.3. Rapid Adaptive Mechanism via Genetics, Epigenetics, and Phenotypic Plasticity

*A. philoxeroides* displays a population genetic structure forged by multiple introduction mechanisms from diverse sources, secondary contact, and regional admixture [15,20,88,89]. Locally, *A. philoxeroides* clonal expansion prevails, while broader landscapes feature mixed lineages forming mosaic patterns, facilitated by transportation hubs, canal networks, and aquaculture corridors that create admixture zones, though individual sites often remain dominated by a few clones [15]. This may yield a “paradox” of metapopulation [90]; that is, low within-site genotypic diversity due to stoloniferous dominance by high-performing clones but contrasted with high among-site and regional diversity shaped by local filters like temperature, hydrology, and nutrients. This metapopulation process can sustain standing variation in the species for swift, context-dependent adaptation without new mutations at invasion fronts. Clonality conceals its diversity, decoupling success from sexual recombination. In addition, the rare sexual events may preserve introduction legacies in the gene pool, detectable only via high-resolution markers rather than phenotypes [39,91,92].

Beyond classical population genetics, *A. philoxeroides* can achieve rapid adaptation through epigenetic mechanisms that connect environmental signals to heritable gene regulation [91,93]. Stressors such as waterlogging, salinity, heavy metals, and pollutants induce DNA methylation and chromatin accessibility shifts [91,94], rewiring pathways for membrane transport, osmolyte production, antioxidant defenses, and morphological changes [83,95]. These epigenetic alterations arise quickly (within growth cycles) and endure across clonal generations, creating an epigenomic “memory” that primes future responses [96]. This particularly underlies variable clone performance through promoter methylation regulates stress genes, histone acetylation controls induction timing, and small RNAs enable post-transcriptional tuning. Thus, the rapid adaptation fits the life history of *A. philoxeroides*, and clonality preserves advantageous epigenotypes intact, while infrequent sexual reproduction reshuffles genetic–epigenetic frameworks, potentially stabilizing adaptive traits under prolonged waterlogging stress.

**Figure 5:**
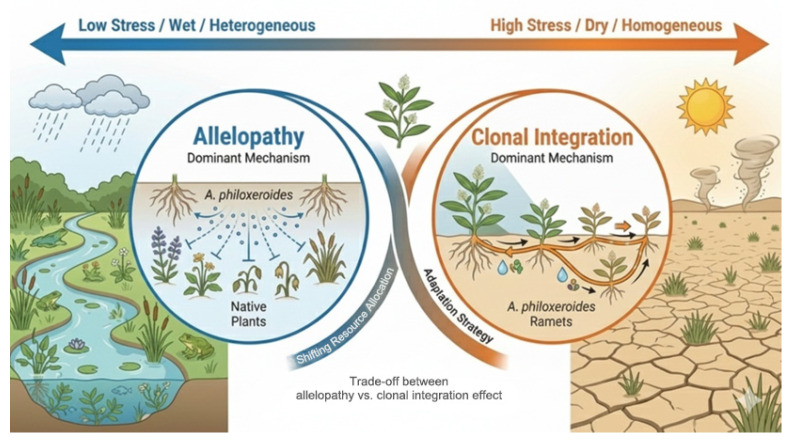
Hypothesized invasion mechanism of *A. philoxeroides*: the trade-off between allelopathy and clonal integration effect. (This illustration was generated using the Gemini 3 Pro Image (Nano Banana Pro) generative AI model).

Moreover, *A. philoxeroides* can also achieve versatile adaptive mechanisms through a synergistic triad of regional genetic mosaics from multiple introductions and admixture, epigenetic “memory” encoding environmental responses, and a flexible metabolome that bridges genotype/epigenotype to fitness, stress tolerance, and community impacts. Metabolomic profiles across hydrological gradients reveal elevated phenolics, quinones, and terpenoids enabling allelopathy, oxidative stress mitigation, and defense while synchronizing with transcriptomic/epigenomic shifts to bolster phenylpropanoid/lignin pathways for stolon strength in nutrient-rich waters, deploy osmoprotectants and ascorbate-glutathione systems under salinity/drought [83,91,97,98]. This process is posited to be a key mechanism behind the invasion of *A. philoxeroides*, highlighting the trade-off between allelopathy and clonal integration along environmental stress gradients (Figure 5).

**Figure 6:**
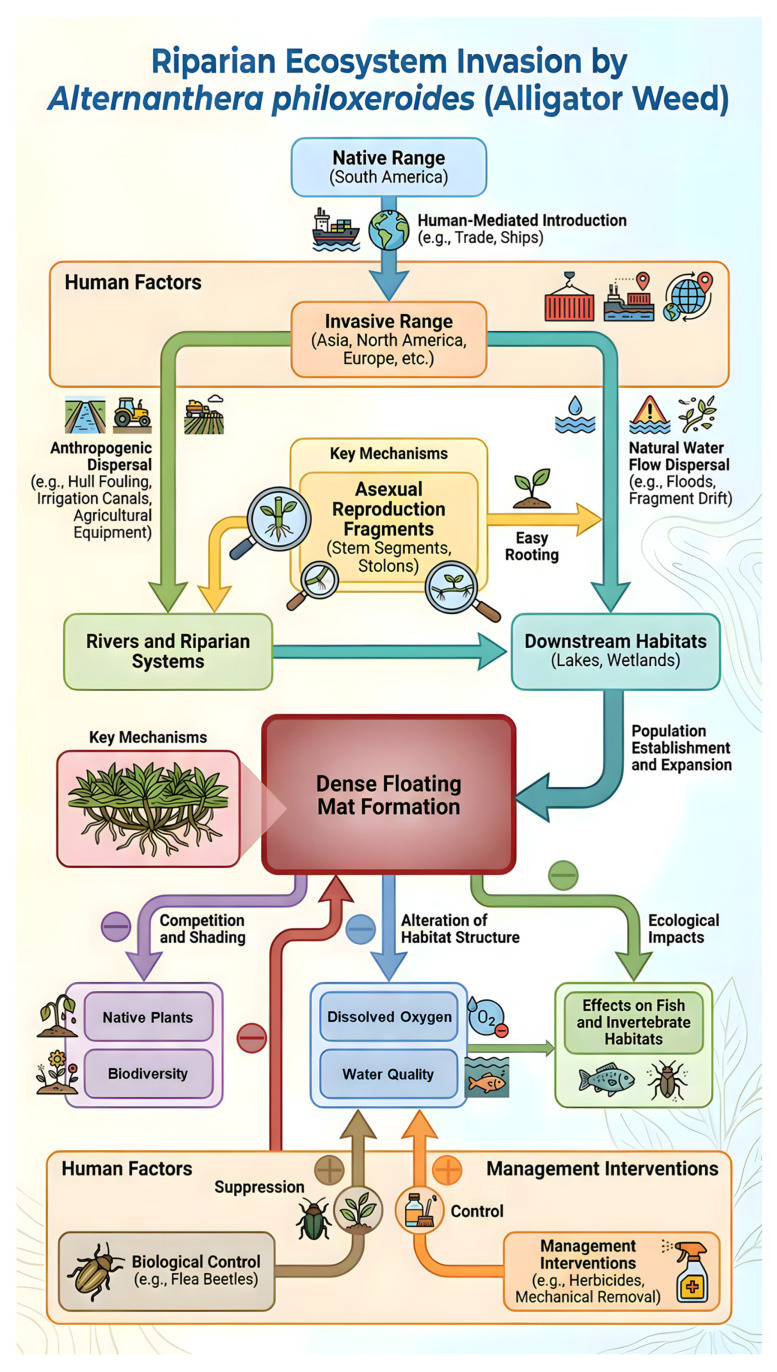
Invasion mechanism, effects, factors, and management by *A. philoxeroides* along the riparian ecosystem. (In the figure, “+” and “−” indicate positive and negative effects, respectively. This illustration was generated using the Gemini 3 Pro Image (Nano Banana Pro) generative AI model).

The phenotypic plasticity of Alternanthera philoxeroides contributes to its invasive potential by allowing rapid morphophysiological responses to environmental variation. These include internode elongation and aerenchyma formation under hypoxia, leaf and stomatal adjustments along light and CO_2_ gradients, shifts in root–shoot allocation in response to nutrient availability, and reversible changes in growth architecture under fluctuating hydrological conditions [20,38]. Beyond self-defense, these compounds foster beneficial microbes for tolerance/efficiency while suppressing natives through canopy shading and exudates [97]. Over invasion timescales, selection canalizes plastic responses via epigenetic stabilization, producing pre-adapted clones resilient to stressors without genetic changes [91,93,96]. Consequently, the overall mechanism by which *A. philoxeroides* invades riparian ecosystems can be characterized as global in nature (Figure 6).

Despite its strong clonal traits, enabling rapid asexual spread and persistence via vegetative fragments, *A. philoxeroides* also shows significant genetic differentiation across invaded ranges. Williams et al. (2020) genotyped 373 plants from 90 U.S. sites via chloroplast markers [15], revealing high [97], geographically structured diversity from multiple introductions rather than local mutations, with distinct haplotypes and greater southern polymorphism tied to founder effects and global admixture. This counters notions of genetic uniformity in clonal invaders, supporting adaptive responses to varied environments through rare sexual reproduction or somatic mutations. Combined with epigenetics, it forms a dual adaptive system: clonality preserves effective epigenotypes, while genetic variation fuels long-term evolution, heightening invasion risks amid climate change.

While many alien species in riparian zones present ecological and economic challenges globally, *A. philoxeroides* distinguishes itself among other invasive flora as a model species for global riparian invasions. This distinction is attributed to its amphibious adaptability, effective clonal propagation through vegetative fragments, and moderate tolerance to drought conditions. These traits allow broad subtropical to warm-temperate spread linked to both hydrological (e.g., floods) and human (e.g., shipping) dispersal, surpassing obligate aquatics such as *Pontederia crassipes* (Mart.) Solms or arid specialists such as *Tamarix ramosissima* Ledeb. (Table 3). It avoids the genetic complexities of high sexual reproduction in species *Reynoutria japonica* Houtt., while mirroring mat-forming impacts (e.g., hypoxic mats reducing biodiversity) of *Ludwigia hexapetala* (Hook. & Arn.) Zardini, Gu & P.H. Raven, and benefits from a century of documented invasions, rich multi-scale data (genomic, remote sensing), and management challenges. Thus, *A. philoxeroides* enables hypothesis testing on source–sink dynamics, climate adaptability, and transferable strategies, making it more tractable and applicable than its comparators with narrower niches or sparser research [33,99].

**Table 3:** Comparison of *A. philoxeroides* with other global major riparian invasive plant species.

Species	Traits/Properties					References
	Origin and Distribution	Growth Form	Reproduction and Dispersal	Environmental Tolerance	Ecological Impact	
*Alternanthera philoxeroides* (Mart.) Griseb.	South America; worldwide in subtropical–warm temperate regions	Amphibious, stoloniferous herb	High clonal spread; sexual reproduction rare	Flooding tolerant, moderate drought tolerance; broad expansion under warming	Dense mats reduce native richness; hypoxia under mats	[20,21,99,100]
*Arundo donax* L.	Eurasia	Tall rhizomatous grass	High clonal spread	Medium flood and drought tolerance; warm temperate–subtropical	Forms monospecific stands that drastically alter riparian structure, increase sedimentation, reduce biodiversity, and create fire hazards	[101,102,103]
*Phragmites australis* (Cav.) Trin. ex Steud.	Eurasia	Tall rhizomatous grass	High clonal spread; sexual reproduction rare	Medium–high tolerance; broad temperate	Alters wetland ecosystems, reduces native plants	[104,105,106]
*Reynoutria japonica* Houtt.	East Asia	Rhizomatous forb/shrub	High sexual reproduction; rhizome fragmentation	Medium tolerance; temperate	Alters riparian communities; dense stands	[107,108]
*Pontederia crassipes* (Mart.) Solms	South America	Free-floating rosette	Very high vegetative spread; sexual reproduction high	Obligate aquatic; tropical–subtropical	Blocks light and water flow, outcompetes submerged macrophytes, and causes persistently low dissolved oxygen under dense mats	[40,109,110]
*Ludwigia hexapetala* (Hook. & Arn.) Zardini, Gu & P.H. Raven	South America	Amphibious, creeping mat-forming	High vegetative spread; sexual reproduction moderate	High flood tolerance, low drought tolerance; warm temperate–subtropical	Surface mats reduce native richness; hypoxia	[111]
*Impatiens glandulifera* Royle	Himalayas	Annual forb	High seed production, vegetative growth	Low–medium flood tolerance; cool–temperate	Alters riverbank soils and vegetation	[112,113]
*Tamarix ramosissima* Ledeb.	Eurasia	Deep-rooted shrub/tree	High sexual reproduction	Very high drought tolerance; arid–semiarid riparian	Alters bank structure, evapotranspiration	[114]

## 3. Integrative Framework of Riparian Invasives Using *A. philoxeroides* as a Model

### 3.1. Distribution Monitoring, Modeling, and Decision Support

Effective and long-term monitoring of riparian invasives requires an integrated infrastructure combining high-resolution monitoring, spatial predictive modeling, and stakeholder-aligned decision-support systems to bridge detection gaps across scales. Remote sensing enables near-real-time surveillance; for instance, the Sentinel-2 multispectral imagery (ESA, Paris, France) (10–20 m) tracks seasonal growth and regrowth of *A. philoxeroides* population in riparian/floodplain habitats [115], and the Planet Scope imagery (Planet Labs PBC, San Francisco, CA, USA) (3–5 m resolution) resolves narrow channels using red-edge/SWIR (Short-Wavelength Infrared) indices and phenology classifiers to differentiate *A. philoxeroides* from other macrophytes [116]. In addition, the Sentinel-1 SAR (Synthetic Aperture Radar; European Space Agency (ESA), Paris, France) offers all-weather detection of floating mats formed by the species population via C-band backscatter [117], and UAVs (Unmanned Aerial Vehicles; e.g., DJI, Shenzhen, China) provide cm-scale orthomosaics and SfM (Structure for Motion) for mat boundaries, canopy, and biomass of the species [118]. Complementing these, eDNA assays detect sparse or hidden parts of the population via water sampling, with metabarcoding and occupancy models enabling basin-scale early detection [119]. For broad scale, cost-effective coverage, IoT (Internet of Things) networks (e.g., attaching GPS modules on the floating mats) and crowdsourcing fuse sensor buoy data with citizen-submitted geotagged photos via apps, allow for the creation of validated early-warning systems for targeted interventions [120,121,122].

Predictive modeling further builds on the monitoring data to forecast optimal intervention points, converting environmental inputs into invasion risk and spread projections. Correlative approaches like MaxEnt (Maximum Entropy Model) and boosted regression trees establish baseline climatic suitability by analyzing occurrence records against environmental variables, defining thermal/moisture limits for current and future scenarios to prioritize surveillance and biosecurity [123,124,125]. Additionally, these approaches have to further account for *A. philoxeroides*’ reliance on aquatic processes (e.g., stem elongation, rooting under shear stress), which prompts to integrate mechanistic models of the model species. These models combine physiological thresholds (including growth, oxygen tolerance, nutrient uptake) with hydrodynamic simulations of flow patterns, water levels, and residence times, ultimately generating maps of high-risk areas for fragment deposition in eddies, embayment, and floodplains during flooding events [126,127]. Furthermore, the predicting models can incorporate human-mediated dispersal by graphing rivers, canals, roads, and trade routes as weighted edges (factoring traffic, biofouling, and floods), simulating metapopulation dynamics to pinpoint super-spreader nodes and evaluate controls like cleaning protocols or ramp closures of riparian invasives.

Based on the distribution models, decision-supporting systems transform the monitoring data and forecasts into actionable plans by optimizing trade-offs among objectives, uncertainties, and constraints, with *A. philoxeroides* serving as an exemplary model for invasive species management. A multi-objective framework evaluates control options (e.g., herbicides, mechanical removal, drawdowns, shading, biocontrol, and pathway blocks) against metrics such as costs, prevented ecosystem losses (e.g., navigation issues, flood impairment, erosion), and non-target risks (e.g., phytotoxicity, bycatch). Pareto optimization reveals efficient trade-offs, while goal programming meets agency targets (e.g., channel widths, budget caps) [128,129]. Spatially and temporally, these systems, leveraging *A. philoxeroides* as a case study, reserve capacity for eDNA/UAV-triggered early detection-rapid response surges and sequence treatments in core areas to disrupt cycles, such as preempting stolon growth or post-thinning herbicides [110,130].

Additionally, uncertainty in decision support can be managed through stochastic optimization and robust decision-making, particularly when *A. philoxeroides* serves as a model invasive species. This involves ensembles of hydrologic and compliance scenarios to stress-test management plans and identify resilient strategies across possibilities. Transparent dashboards display assumptions, data sources, and confidence intervals, allowing managers and stakeholders to explore scenarios, adjust priorities (e.g., prioritizing fishery protection during spawning), and implement adaptive cycles where outcomes update models, retrain classifiers, and refine cost-effectiveness. This holistic loop (encompassing ongoing sensing, physics- and network-driven forecasts, and uncertainty-informed optimization) shifts from reactive to proactive management, optimizing resources to reduce propagule pressure, seal off dispersal routes, and erode *A. philoxeroides*’ dominance in aquatic habitats.

### 3.2. A Paradigm for Global Dispersal Pathways Modeled by A. philoxeroides

Building on the distribution monitoring systems, which incorporate ground-truth validation through physical field sampling to ensure the accuracy of remote sensing and other detection methods, a paradigm can be proposed to advance the study of *A. philoxeroides* global dispersal pathways through AI-driven analysis on a dynamic knowledge graph. This paradigm supports ongoing evidence collection and targeted interventions, centered on multi-source attribution, which integrates genome profiling to trace introductions, admixture, and clonality; herbarium records, trade manifests, and port logs for historical routes; vessel tracks for maritime links; flood/storm data for hydrochory; and remote sensing from the monitoring system.

**Figure 7:**
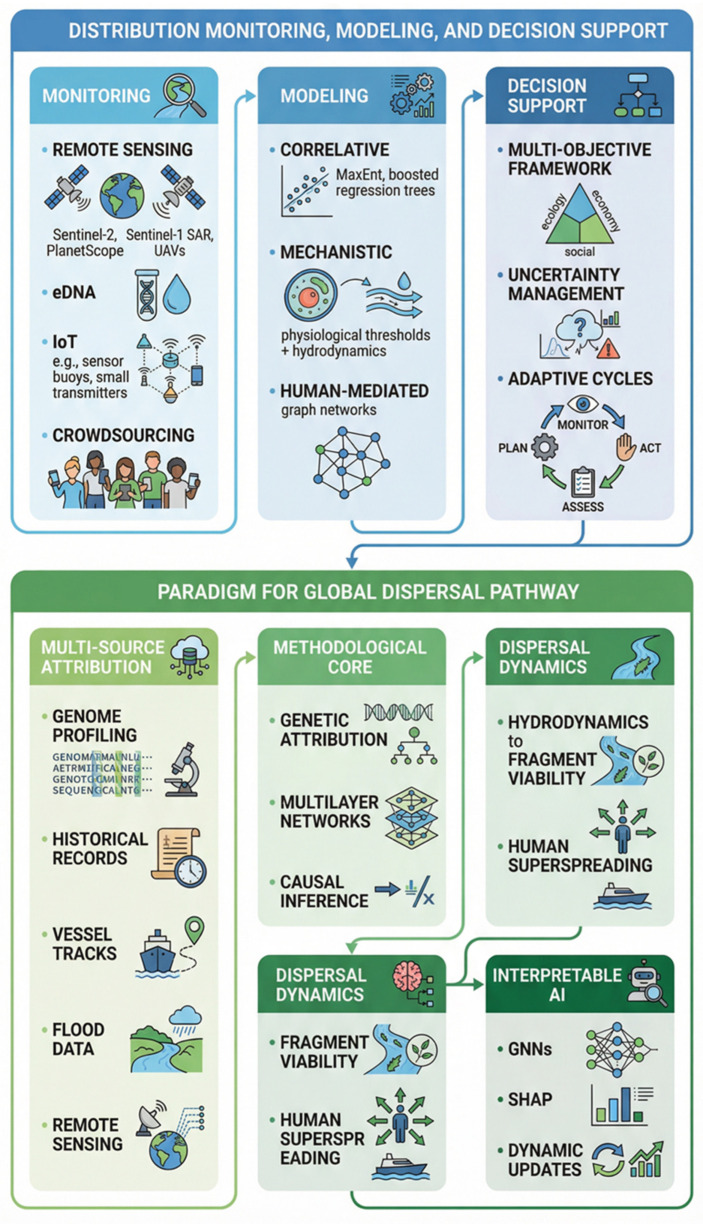
The integrative framework of riparian invasives using *A. philoxeroides* as a model. (This illustration was generated using the Gemini 3 Pro Image (Nano Banana Pro) generative AI model).

The methodological core of this proposed paradigm is a three-tier stack: (1) genetic attribution uses approximate Bayesian computation, coalescent graphs, and phylogeographic embeddings to assess colonization and admixture [131]; (2) multilayer networks connect trade, transport (maritime/riverine/road/rail), hydrology, and environmental layers with minimum spanning trees for routes, random-walk betweenness for hubs, and time-aware paths for seasonal factors [132,133]; and (3) causal inference evaluates policy impacts via regression discontinuity, difference-in-differences, and synthetic controls to separate effects from confounders like climate or economic shifts [134] (Figure 7).

In particular, dispersal dynamics of *A. philoxeroides* shall link hydrodynamics to fragment viability in riparian invasives, with thresholds for rooting in low shear, residence times, drawdown effects on strandlines/oxygen, and turbulence promoting entrapment [135]. Human super-spreading (e.g., aquarium dumps, dredge moves) has to be modeled with heavy-tailed distributions and extreme value theory for outliers [136,137,138]. Thus, dispersal pathway dynamics of the model species fuse eDNA, multispectral cover, traffic, and hydrology data via hierarchical Bayesian methods, producing probabilistic presence maps with uncertainty and spatial/temporal tracking [139,140,141].

Furthermore, an interpretable AI on the knowledge graph unifies these analytic processes, using GNNs (Graph Neural Networks) and time embeddings for predictions, with SHAP (Shapley Additive Explanations)/counterfactuals identifying drivers (e.g., lock operations) and leverage points (e.g., dredge protocols) to disrupt pathways [141]. The paradigm operates dynamically, updating with new data to re-evaluate routes and risks. Exportable to similar invasives, it emphasizes unified genetics, networks, and mechanisms for attributing and interrupting dispersal at scale.

## 4. Bridging Invasion Ecology and Riverscape Management via the Model Species

Bridging invasion of *A. philoxeroides* and riverine management at global scale requires a more advanced integrative program linking micro-level biological mechanisms to meso-scale landscapes and macro-scale human systems. At the micro scale, priorities include unraveling gene–epigene–metabolite–microbiome interactions for adaptations like waterlogging tolerance of the model species, using multi-omics in stress experiments, CRISPR (Clustered Regularly Interspaced Short Palindromic Repeats) gene editing tools, causal network inference, and common garden transplants to identify regulatory feedbacks, thresholds, and fitness effects for model integration [142,143,144,145,146].

At the meso scale, the micro scale shall integrate into process-based models which simulate hydrodynamics, drawdown effects, and urban infrastructure on establishment and spread. Also, the integrated process-based models can further incorporate the model species’ fragment transport, rooting probabilities, mat feedback, and validation through controlling experiments and metapopulation models with real-time data (eDNA, UAV, SAR) for forecasting and prioritization [147,148,149,150,151].

**Figure 8:**
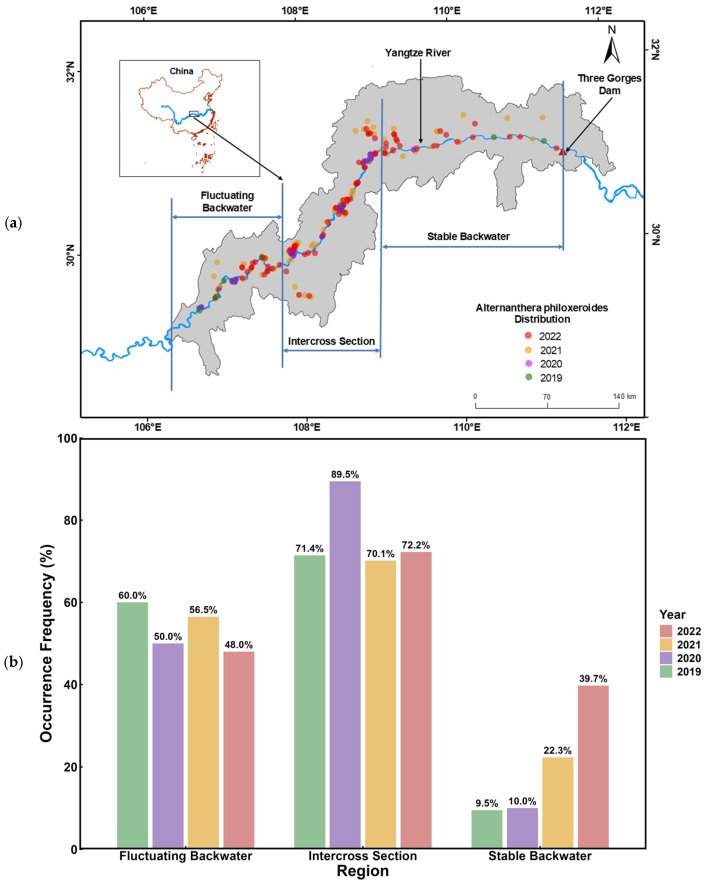
Distribution and occurrence frequency of *A. philoxeroides* from 2019 to 2022 in different backwater zones formed by regulation of the Three Gorges Reservoir: (**a**) distribution; (**b**) occurrence frequency.

Dammed rivers serve as both conduits for biological invasions and valuable arenas for investigating meso-scale processes. In the case of the Three Gorges Dam Reservoir on China’s Yangtze River, a prominent dam-induced modification in the upstream river involves the generation of a backwater effect within the reservoir [152]. This phenomenon establishes stable backwater zones proximate to the dam, fluctuating backwater zones at greater distances, and transitional intercross zones between the stable and fluctuating backwater. Such discontinuous river flow regimes closely align the invasion pathways of *A. philoxeroides* with control-oriented management practices under reservoir operations. In this area, our time-series field data spanning for 4 years (Figure 8) revealed distinct spatiotemporal patterns of *A. philoxeroides* invasion along the riparian zone of the reservoir. From 2019 to 2022, occurrence frequencies consistently peaked at the intercross section of reservoir backwater at range from 70.1% to 89.5%, remained moderate at the fluctuating backwater section from 48.0% to 60.0%, and showed a marked escalation at the stable backwater section from 9.5% to 39.7%, underscoring the reservoir’s role in creating an “invasion highway” along the non-continuum of the dammed river, with the intercross section of reservoir backwater emerging as the primary hotspot for *A. philoxeroides* (and other alien species) proliferation [153]. Integrating these patterns with concomitant hydrological records, such as water level fluctuations and flow velocities, provides empirical benchmarks for parameterizing establishment probabilities as functions of hydrodynamic conditions across reservoir sectors. This integrative modeling approach can facilitate validation against observed invasion intensities, thereby pinpointing high-risk zones for targeted management interventions.

At the macro scale, models developing from the model species *A. philoxeroides* must address dynamic global networks influenced by trade, tourism, geopolitics, and climate, using time-varying multiplex graphs from customs and tourism data for scenario analyses of extreme events, super-spreaders, and interdictions, coupled with hydroclimate projections [154,155]. Supporting this is an open data infrastructure with standardized repositories for remote sensing, eDNA, site, and treatment data, plus interoperable ontologies and reproducible pipelines [156,157]. In addition, methodological shifts emphasize interventions via optimal control, reinforcement learning for tactic sequencing, and multilayer network analysis to detect cascades, thresholds, and hotspots for robust optimization under uncertainty [158].

To effectively integrate the approaches at multiple scales for managing invasives, it is crucial to establish a proactive hub or platform that brings them together. This hub will leverage remote sensing and high-resolution satellite imagery for rapid assessment of invasion patterns and extent, while also deploying AI-guided autonomous vehicles for accurate mapping and removal [159,160,161]. The resulting invasive biomass, rather than being discarded, can be safely converted into biogas or biochar under established safety protocols [162], supporting both waste reduction and resource recovery. Effective governance requires adaptive experimental frameworks to evaluate management effectiveness [163,164] and economic incentives that encourage stakeholder participation and adherence to best practices [165]. Furthermore, international cooperation through cross-border agreements [166], combined with interpretable analytics powered by knowledge graphs to ensure transparency, can significantly enhance overall management efficacy.

By shifting from reactive suppression to anticipatory control, this strategic framework prioritizes the minimization of propagule pressure, blockage of dispersal pathways, ecosystem restoration, and the fortification of resilience amidst environmental changes. The inherent traits of *A. philoxeroides*, such as its adaptability, broad environmental tolerance, and robust propagation mechanisms, serve as significant strengths in understanding global invasive patterns. This allows for the formulation of targeted management strategies that are adaptive, predictive, and ultimately transformative for ecosystems impacted by invasive species.

## 5. Key Challenges for Future Perspectives

Achieving anticipatory, network-driven management of *A. philoxeroides* requires addressing a series of interconnected challenges related to data, dynamics, ethics, and scientific methodologies—transforming these obstacles into avenues for innovation. Persistent observation bias and “silent evidence” continue to undermine efforts, as detections are often concentrated in easily accessible locations, neglecting remote areas, which results in distorted models and false absences [167]. To mitigate this issue, it is essential to implement stratified sampling techniques and standardized protocols for eDNA and imagery, which can help confirm negative detections [168]. Additionally, employing detectability-adjusted occupancy models will enhance the reliability of risk mapping and assessments. These challenges are further exacerbated by non-stationarity—driven by climate change, evolving infrastructure, and shifting global trade routes—which continuously alters the drivers and patterns of biological invasions [169]. This can be addressed through online learning methods that facilitate dynamic updates, ensemble scenario testing, and change-point detection to ensure that strategies remain relevant and adaptable [170,171].

The complexity of eco–evolutionary feedback introduces additional challenges, as rapid adaptations, such as epigenetic plasticity, can broaden ecological niches and reduce the effectiveness of control measures. Furthermore, vigorous interventions may inadvertently favor the selection of resistant clones [172]. To tackle these issues, it is crucial to incorporate evolutionary principles into management models by connecting traits, selection dynamics based on hydrology and intervention tactics, and establishing balanced strategies that avoid creating directional pressures.

Ethical governance also plays a vital role in managing non-target risks, particularly when utilizing genetic tools that raise concerns around biosafety and equity. Strategies should prioritize reversible methods, implement phased trials with oversight, develop clear exit strategies, and actively involve communities to ensure fair outcomes. Lastly, promoting reproducibility and open science will foster trust across international boundaries, necessitating comprehensive metadata, open-source code, and timely sharing of data with persistent identifiers to facilitate verification, comparability, and collaborative progress. Addressing these challenges will reduce the likelihood of misleading models, unsuccessful interventions, and erosion of trust, ultimately enabling effective disruption of invasion pathways and aiding ecosystem recovery.

Despite these obstacles, there are significant opportunities for innovative advancements. Utilizing high-resolution genomic techniques, such as whole-genome resequencing, haplotype networks, and approximate Bayesian computation, can greatly enhance our ability to reconstruct introduction pathways, quantify propagule pressure, and elucidate source–sink dynamics [173]. Additionally, developing comprehensive, geo-referenced datasets that incorporate bias-corrected sampling protocols will support extensive sampling across ranges, allowing for global comparisons, robust modeling, and accurate estimates of spread. By integrating pathway models with eco–evolutionary dynamics, researchers can investigate how gene flow through dispersal influences niche shifts, employing a framework that combines transport networks, environmental suitability, and trait evolution to predict potential expansion or genetic swamping. These enhancements are poised to increase the novelty and real-world applicability of the study.

## 6. Conclusions

Invasion of *A. philoxeroides* stands as a compelling model for understanding the global dispersal pathways of invasive species, due to its intricate invasion history characterized by multiple introductions, genetic admixture, clonal propagation, and significant anthropogenic facilitation. The path forward for managing this invasive species—and others like it—requires a concerted effort to innovate at every level of investigation and intervention.

Key to advancing our understanding and management of invasive species is the integration of diverse data sources and cutting-edge analytical techniques. By harnessing multi-source, spatiotemporal evidence, we can employ rigorous causal analytics that illuminate not just the where and how of dispersal, but also the underlying mechanisms that mandate such invasions. Coupling sophisticated hydrodynamic models with real-world landscape processes allows for the anticipation of invasion dynamics, leading to proactive, rather than reactive, riverscape management strategies.

Moreover, institutionalizing transparency through open, cross-national data collaborations and reproducible research practices will create a robust framework for tracking invasives. Innovations in population genomics, coupled with remote sensing technologies, eDNA analysis, big data technology and the mapping of transport networks, will enable us to pinpoint introduction sources, identify dominant invasion routes, and quantify the leverage points for effective intervention with defensible uncertainty.

The confidence in these approaches is grounded in their applicability. The methodologies developed around *A. philoxeroides* can be scaled to inform management protocols for other aquatic and terrestrial invaders. By embracing this integrative framework, focusing on multi-evidence attribution, interpretable causal inference, and physics-informed experimentation, we can disrupt invasion pathways decisively.

Ultimately, our commitment to these innovative strategies promises not only to enhance ecological integrity but also to yield significant socio-economic benefits through the effective management of invasive species. This synthesis of insights places us on a clear trajectory toward establishing resilient ecosystems and informed policy frameworks, ensuring that we are prepared for the challenges of global biodiversity conservation amidst a rapidly changing environment.

## Data Availability

The raw data supporting the conclusions of this article will be made available by the authors on request. The data are not publicly available due to the ongoing long-term monitoring of the study sites and planned follow-up analyses based on the same dataset.
